# Exogenous lipid pneumonia related to long-term use of Vicks VapoRub® by an adult patient: a case report

**DOI:** 10.1186/s12901-016-0032-6

**Published:** 2016-08-19

**Authors:** I. Cherrez Ojeda, J. C. Calderon, J. Guevara, D. Cabrera, E. Calero, A. Cherrez

**Affiliations:** 1 Universidad de Especialidades Espiritu Santo, School of Medicine, Samborondón, Guayas, 09150 Guayaquil, Ecuador; 2 Respiralab, Respiralab Research Group, Guayaquil, Ecuador; 3 Interhospital, Guayaquil, Ecuador; 4 University of Heidelberg, School of Medicine, Heidelberg, Germany

**Keywords:** Exogenous lipoid pneumonia, Rhinitis, Petroleum jelly, Case report

## Abstract

**Background:**

Use of petroleum-based over the counter remedies such as Vicks VapoRub to alleviate symptoms of rhinitis is common and can be effective, but carries under-appreciated risks of adverse side effects. In this case report we highlight Exogenous Lipoid Pneumonia (ELP), an uncommon condition that results from accumulation of exogenous lipids in the alveoli, as an adverse side effect of long-term Vicks VapoRub use.

**Case presentation:**

We present the case of an 85-year-old female patient with ELP apparently due to continuous application of Vicks VapoRub® to her nostrils to alleviate chronic rhinitis. She was diagnosed incidentally via chest radiograph and computed tomography (CT) scan done as follow up to finding elevated C-reactive Protein during a routine exam. The CT scan revealed a pulmonary consolidation in the lower lobe of the right lung with fat density combined with low density areas associated with focal ground-glass opacities. The patient was advised to discontinue use of petroleum-based products, and was prescribed intranasal corticosteroids for her rhinitis. Follow up 2 years later showed that the lipid consolidation had diminished in size by approximately 10 %.

**Conclusion:**

Physicians must be aware that ELP can develop as a result of long-term application of petroleum-based oils and ointments to the nose and discourage such use of these products. Patients who have used petroleum-based products in this way should be screened for ELP. CT scan is the best imaging modality for establishing the diagnosis. The treatment of this condition is not well defined, but, as shown in this case, the size of the lipid mass can decrease after use of petroleum based substances is discontinued.

**Electronic supplementary material:**

The online version of this article (doi:10.1186/s12901-016-0032-6) contains supplementary material, which is available to authorized users.

## Background

Exogenous lipoid pneumonia (ELP) is a rare condition resulting from the aspiration or inhalation of fat-like material of animal, vegetable or mineral origin [1]. The frequency of ELP is difficult to establish, but autopsy series have revealed frequencies of 1 to 2.5 % [2]. Forty four cases of ELP were identified in a nationwide retrospective study in France between 1981 and 1993 [3]. The principal factor for ELP is the inhalation of inert, long-chain, saturated hydrocarbons found in petroleum. Mineral oils and ointments can inhibit the cough reflex and ciliary motility, thus facilitating inhalation. Their presence in the pulmonary parenchyma causes a foreign body type of inflammatory reaction [4, 5]. Most ELP cases result from the use of oil-based laxatives for the treatment of constipation, or from nasal instillation of oily products, including petroleum ointment products such as Vaseline or Vicks VapoRub® [6], for relief of chronic rhinopharyngeal diseases. Reported sources also include lip balm/gloss [7]. Most of the patients who aspirate these substances are elderly people who have difficulty swallowing due to anatomic or functional issues, and who have a history of topical application or ingestion of lipid products [5].

Individuals with ELP can present with unspecified symptoms such as cough, dyspnoea, chest pain, haemoptysis or fever. They can also be asymptomatic, in which case ELP may be identified as an incidental abnormality on radiologic imaging [5, 8]. On physical examination findings are usually normal, although dullness on percussion, crackles, wheezes or rhonchus may be found [4]. Laboratory findings such as leucocytosis and increased erythrocyte sedimentation rate can be found, especially when complicated by an infection. In one study increased sedimentation rate was observed in 61 % of ELP cases [3]. Pulmonary function test results have shown a restrictive pattern in long-standing disease [4]. Prominent radiologic abnormalities have been found in the absence of symptoms or clinical signs in many ELP cases [9].

High-resolution computed tomography (CT) is the best imaging modality for establishing the diagnosis of ELP. The most frequent findings are airspace consolidations, ground-glass opacities, interlobular septal thickening, airspace nodules (small poorly-defined centrilobular nodules), and mass-like lesions [10]. The mass is typically irregular or spiculated as a result of chronic inflammation and secondary fibrosis. Since its clinical and radiological presentations are nonspecific, ELP may mimic many other diseases, including lung tumours [11]. The presence of fat in the mass is a diagnostic feature of ELP [12]. The radiologic manifestations of ELP can improve slowly over time, but typically remain stable even if exposure to oils or fats is discontinued. ELP-related fibrosis and destruction of normal lung architecture can result in cor-pulmonale [13].

Patients with rhinitis, a condition that usually presents with nasal congestion, rhinorrhea, sneezing and itching, often self-medicate, using over-the-counter decongestants or folk remedies [14, 15]. One traditional folk remedy consists of instilling medicated oil or ointment into the nose and sniffing it. A variety of oil-based products have been used, including pure sesame oil [12] and mentholated petroleum-based ointments such as Vicks VapoRub®.

Here we report the case of an patient in a routine medical visit with history of rhinitis and mentholated mineral ointment use who was found to have ELP.

## Case presentation

An 85-year-old Ecuadorian female self-referred for her annual routine visit to family physician (Additional file 1: Timeline Table). She had history of recurrent allergic rhinitis events with nocturnal runny nose since childhood. She revealed daily use of the over-the-counter mentholated mineral ointment decongestant Vicks VapoRub® for approximately 50 years to ease her discomfort. She applied this product to her chest, palms and feet, and aspirated it through her nose.

The patient had no signs or symptoms of respiratory disease at the time of the visit. Her physical examination was unremarkable. Her medical history included type 2 diabetes mellitus and hypertension.

**Fig. 1:**
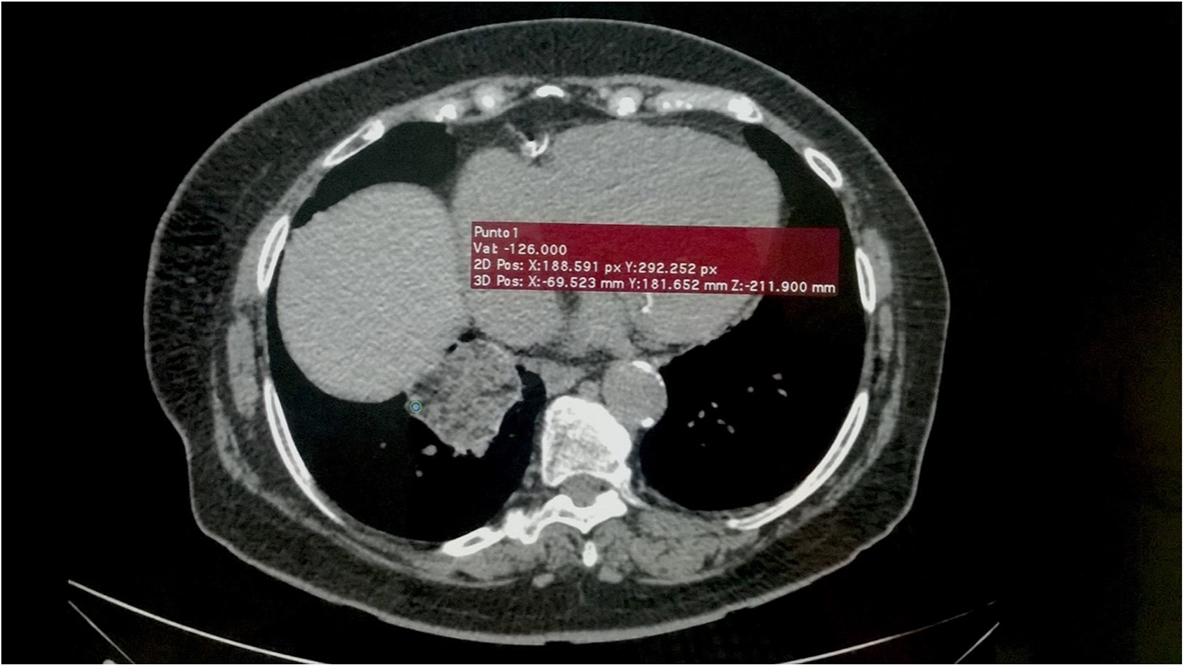
Thoracic CT at the first medical consultation

Laboratory findings demonstrated an increased CRP (31.5 mg/L, reference value 0.0 – 5.0 mg/L). After obtaining no relevant results, a follow up chest radiograph was ordered. It showed airspace consolidation as an irregular mass-like-lesion in the right lower lobe. The patient continued to be asymptomatic despite the presence of this mass. A thoracic computed tomographic (TCT) scan of the chest was ordered. This showed a pulmonary consolidation of 5.0 × 4.5 cm in the posterior basal segment of the lower lobe of the right lung, containing negative density regions measuring between −130 HU and −61 HU, indicative of intrapulmonary lipid (Fig. 1). Focal ground-glass opacities were observed in the periphery and scattered ground glass opacities bilaterally, which are suggestive of pneumonitis. We recommended that the patient stop using Vicks VapoRub®, and prescribed intranasal corticosteroids for her rhinitis.

**Fig. 2:**
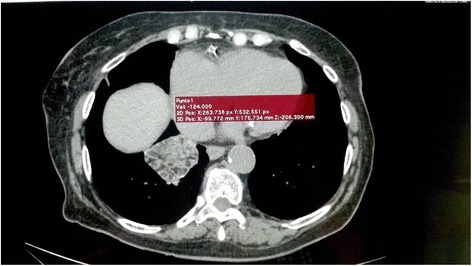
Thoracic CT 26 months after cessation of petroleum jelly

Twenty-six months after stopping daily mentholated ointment application a follow-up TCT and CRP was ordered. The pulmonary consolidation described above was still apparent, but the bilateral scattered ground glass opacities had diminished and the size of the mass had decreased by 0.5 cm in each dimension (4.5 × 4.0 cm) (Fig. 2). CRP was in reference value (2.03 mg/L)

## Discussion

Although ELP is an unusual cause of chronic lung disease, it is an important consideration in the differential diagnosis of several pulmonary syndromes because progression appears to be halted, or at least slowed, by stopping exposure to the offending lipid substance. In our patient the presence of negative TCT density values in the mass implied the presence of lipid. That fact, together with follow up scan results, allowed us to rule out malignancy.

Several authors have suggested that negative density values between −150 and −30 HU in areas of consolidation are highly suggestive of intrapulmonary fat, and are consistent with ELP, especially when associated with a history of exposure to mineral oil or ointment. In order to prevent a false positive interpretation, measurements should be taken in the most hypodense part of the consolidation area, free of any aerated parenchyma on the periphery or areas of air bronchogram [5, 16, 17].

Systemic steroids have been used to slow the inflammatory response associated with ELP, but their use is supported only by few anecdotal reports [18–21]. Because inflammation will resolve spontaneously with cessation of exposure in most cases, it seems steroids can be withheld unless the lung injury is severe and progressive [8, 19].

ELP is often mild and does not appear to progress if use of the causative agent is stopped. However, there are few studies in which ELP masses are followed and measured after patients have stopped using oil-based substances. In one case involving a 38 year old woman with ELP, the mass was reported to have diminished 2 years after stopping oil ingestion, but the decrease was not quantified [22]. In the present case we measured the ELP mass at the time of diagnosis and 26 months after the patient discontinued use of mentholated petroleum ointment, and found a 10 % decrease in size. Prospective studies should be done to determine the average rate at which ELP masses decrease after use of the causative agent is stopped.

The best treatment for ELP is prevention. However, the fact that ELP is a potential risk associated with chronic use of lipid substances is not always appreciated, even by physicians. A recent review about rhinitis in geriatric populations said that use of oil substances in the nose is generally safe and can be used adjunctively with other treatment [23]. Mineral oil and ointment products are sold without prescription, and no information is provided for consumers or clinicians on possible hazards, especially among people at risk of aspiration, including the elderly and people with gastroesophageal reflux, dysphagia, nasal congestion or neurologic disease. Mentholated ointments have been shown to be cilio-toxic and mildly proinflammatory, increasing mucus secretion while decreasing mucus clearance [24]. This gives the sensation of increased airflow and therefore relief of symptoms, but in fact there is no improvement in airflow or decrease in nasal resistance with the use of these products [25].

Health professionals need to be aware of the risks and discourage the uncontrolled use of mineral oil and ointment, especially for the very young and the elderly [26].

## Conclusion

We suspect that our patient developed ELP due to excessive use of Vicks VapoRub® for chronic rhinitis. ELP was confirmed by TCT, which was ordered because of elevated CRP findings. Physicians need to be aware of this chronic adverse effect and discourage the use of mineral oil and ointment. The ability to recognize radiological manifestations of ELP can help establish an early diagnosis and start timely intervention.

The main intervention is discontinuing use of lipid substances. In the reported case, we found a 10 % decrease in size of the ELP mass 26 months after the patient stopped using Vicks VapoRub®. Further studies should be done to determine average rates at which ELP-related masses decrease after use of lipid substances is stopped.

## Additional file

Additional file 1: Timeline Table. (DOCX 17 kb)

## Abbreviations

CRP: C-Reactive Protein
CT: Computed Tomography
ELP: Exogenous Lipoid Pneumonia
TCT: Thoracic Computed Tomographic

## References

[1] Betancourt SL, Martinez-Jimenez S, Rossi SE, Truong MT, Carrillo J, Erasmus JJ. Lipoid pneumonia: spectrum of clinical and radiologic manifestations. Am J Roentgenology American Roentgen Ray Society. 2012. 10.2214/AJR.09.3040 20028911

[2] Rouffy J, Almosni MCN (1976). Aspects actuels des lipoidoses pulmonaires exogènes de l’adultee. Ann Med Interne.

[3] Gondouin A, Manzoni P, Ranfaing E, Brun J, Cadranel J, Sadoun D (1996). Exogenous lipid pneumonia: a retrospective multicentre study of 44 cases in France. Eur Respir J.

[4] Hadda V, Khilnani GC (2010). Lipoid pneumonia: an overview. Expert Rev Respir Med.

[5] Baron SE, Haramati LB, Rivera VT (2003). Radiological and clinical findings in acute and chronic exogenous lipoid pneumonia. J Thorac Imaging.

[6] Brown AC, Slocum PC, Putthoff SL, Wallace WE, Foresman BH (1994). Exogenous lipoid pneumonia Due to nasal application of petroleum jelly. Chest American College of Chest Physicians.

[7] Becton DL, Lowe JE, Falletta JM (1984). Lipoid pneumonia in an adolescent girl secondary to use of lip gloss. J Pediatr.

[8] Simmons A, Rouf E, Whittle J (2007). Not your typical pneumonia: a case of exogenous lipoid pneumonia. J Gen Intern Med.

[9] Marchiori E, Zanetti G, Mano CM, Hochhegger B (2011). Exogenous lipoid pneumonia. Clinical and radiological manifestations. Respir Med.

[10] Bell MM (2015). Lipoid pneumonia: an unusual and preventable illness in elderly patients. Can Fam physician Médecin Fam Can.

[11] Bréchot JM, Buy JN, Laaban JP, Rochemaure J (1991). Computed tomography and magnetic resonance findings in lipoid pneumonia. Thorax.

[12] Johnsen J, Bratt BM, Michel-Barron O, Glennow C, Petruson B (2001). Pure sesame oil vs isotonic sodium chloride solution as treatment for dry nasal mucosa. Arch Otolaryngol Head Neck Surg.

[13] Chin NK, Hui KP, Sinniah R, Chan TB (1994). Idiopathic lipoid pneumonia in an adult treated with prednisolone. Chest.

[14] Demoly P, Allaert F-A, Lecasble M (2002). ERASM, a pharmacoepidemiologic survey on management of intermittent allergic rhinitis in every day general medical practice in France. Allergy.

[15] Maurer M, Zuberbier T (2007). Undertreatment of rhinitis symptoms in Europe: findings from a cross-sectional questionnaire survey. Allergy.

[16] Tahon F, Berthezène Y, Hominal S, Blineau N, Guérin J-C, Cinotti L (2002). Exogenous lipoid pneumonia with unusual CT pattern and FDG positron emission tomography scan findings. Eur Radiol.

[17] Descatha A, Mompoint D, Ameille J (2006). Occupational paraffin-induced pulmonary fibrosis: a 25-year follow-up. Occup Med (Lond).

[18] Shaikh AY, Oliveira PJ (2014). Exogenous lipoid pneumonia (Fire-eater’s Lung). Am J Med.

[19] Kuroyama M, Kagawa H, Kitada S, Maekura R, Mori M, Hirano H (2015). Exogenous lipoid pneumonia caused by repeated sesame oil pulling: a report of two cases. BMC Pulm Med.

[20] Álvarez-Cordovés MM, Mirpuri-Mirpuri PG, Rocha-Cabrera P, Pérez-Monje A (2013). Neumonía lipoidea: a propósito de un caso. Semergen.

[21] Yampara Guarachi GI, Barbosa Moreira V, Santos Ferreira A, Sias SM, Rodrigues CC, Teixeira GH (2014). Lipoid pneumonia in a gas station attendant. Case Rep Pulmonol.

[22] Doubková M, Doubek M, Moulis M, Skřičková J (2013). Exogenous lipoid pneumonia caused by chronic improper use of baby body oil in adult patient. Rev Port Pneumol.

[23] Pinto JM, Jeswani S (2010). Rhinitis in the geriatric population. Allergy Asthma Clin Immunol BioMed Central Ltd.

[24] Abanses JC, Arima S, Rubin BK (2009). Vicks VapoRub induces mucin secretion, decreases ciliary beat frequency, and increases tracheal mucus transport in the ferret trachea. Chest.

[25] Burrow A, Eccles R, Jones AS (1983). The effects of camphor, eucalyptus and menthol vapour on nasal resistance to airflow and nasal sensation. Acta Otolaryngol.

[26] Human S. Administration for community living. Administration on Aging. 2016.

