# Supplementary material for: Exogenous lipid pneumonia related to long-term use of Vicks VapoRub® by an adult patient: a case report
Source: BMC Ear Nose Throat Disord. 2016 Aug 19;16:11. doi: 10.1186/s12901-016-0032-6 (PMC4992226; doi:10.1186/s12901-016-0032-6)
Supplement: Additional file 1: — Timeline Table. (DOCX 17 kb) [file 12901_2016_32_MOESM1_ESM.docx]

**Timeline Table**

| **Dates** | **Relevant Past Medical History and Interventions** | | | |
| --- | --- | --- | --- | --- |
| October 29, 2013 | Recurrent allergic rhinitis, type 2 diabetes, hypertension. | | | |
| **Date** | **Summaries from Initial and Follow-up Visits** | **Diagnostic Testing**  **(including dates)** | **Interventions** |  |
| October 29,  2013 | No signs or symptoms of respiratory disease.  Increased C-reactive Protein (31.5 mg/L, reference value 0.0 – 5.0 mg/L). | Physical examination (October 29, 2013)  Blood test (October 29, 2013) |  |  |
| October  30,  2013 | Pulmonary consolidation of 5.0 x 4.5 cm in the posterior basal segment of the lower lobe of the right lung, containing negative density regions measuring between -130 HU and -61 HU. | Thoracic computed tomographic (TCT) scan (October 30, 2013) |  |  |
| October 31,  2013 | Diagnosis of Exogenous Lipoid Pneumonia. | Diagnosis (October 31, 2013) | Stop using Vicks VapoRub®  Prescribe intranasal corticosteroids for her rhinitis |  |
| January 4,  2014 | Follow-up visit:   - Patient reported stopping the use of Vicks VapoRub ® | Physical examination (January 4, 2014) | Recommend continue stopping the use Vicks VapoRub®  Prescribe intranasal corticosteroids for her rhinitis |  |
| January 8,  2016 | Final Follow-up visit:  . Consolidation was still apparent, but bilateral scattered ground glass opacities had diminished and the size of the mass had decreased by 0.5 cm in each dimension.  C-reactive Protein in normal value (2.03 mg/L) | Thoracic computed tomographic (TCT) scan ( January 8, 2016) |  |  |
| January 8,  2016 | Final outcome for this episode of care |  |  |  |
